# Race and the Fragility of the Legal Distinction between Juveniles and Adults

**DOI:** 10.1371/journal.pone.0036680

**Published:** 2012-05-23

**Authors:** Aneeta Rattan, Cynthia S. Levine, Carol S. Dweck, Jennifer L. Eberhardt

**Affiliations:** Department of Psychology, Stanford University, Stanford, California, United States of America; University of Bologna, Italy

## Abstract

Legal precedent establishes juvenile offenders as inherently less culpable than adult offenders and thus protects juveniles from the most severe of punishments. But how fragile might these protections be? In the present study, simply bringing to mind a Black (vs. White) juvenile offender led participants to view juveniles in general as significantly more similar to adults in their inherent culpability and to express more support for severe sentencing. Indeed, these differences in participants’ perceptions of this foundational legal precedent distinguishing between juveniles and adults accounted for their greater support for severe punishment. These results highlight the fragility of protections for juveniles when race is in play. Furthermore, we suggest that this fragility may have broad implications for how juveniles are seen and treated in the criminal justice system.

## Introduction

The U.S. is a world leader in punitiveness. Research has documented that the U.S. applies harsher penalties and incarcerates more of its adult populace (for longer periods of time) than any other industrialized, democratic nation in the world [1]-[3]. Despite the trend of increasing punitiveness in the adult criminal justice context, one class of individuals has been consistently protected: juveniles. As a general rule, the law considers juvenile offenders to be less culpable than adults, and for this reason juveniles cannot merit punishments as severe as those available for adults, even for the same crime [4], [5].

Although these protections have existed for some time, their application to severe offenses has been more recent [4], [5]. When juveniles commit serious violent crimes, this protection may seem at odds with the goal of meting out punishment appropriate to the severity of the offense. In other words, when juveniles commit “adult” enough crimes, there may seem to be a justifiable basis for assigning them adult punishments. Indeed, this argument was evident in the debate before the Supreme Court over whether life in prison without the possibility of parole, the most severe punishment available for juveniles, ought to remain legal for non-homicide cases. Although the Court ultimately determined that juveniles’ reduced standard of culpability should protect them from such severe sentencing in non-homicide cases, the Justices issued a split 5-4 decision [5], suggesting that some of the Justices may have been more swayed by the “adult time for adult crime” argument than the established protection associated with juveniles.

Given how recent this protection is in the context of severe offenses, might a heightened desire to punish weaken it? One factor that has been reliably shown across justice contexts to inappropriately heighten people’s desire for severe punishments is race. Black American adults are incarcerated at a higher rate than White Americans [1], [3], [6] and are disproportionately likely to receive severe sentences such as the death penalty [7]. Research has even shown that the more “Black” an adult offender is perceived to be, the greater their likelihood of being sentenced to death [8]. Moreover, Black juveniles who are transferred to adult court for trial and sentencing receive significantly more punitive sentences than White juveniles, and this practice is on the rise [9].

Extending this past research, we systematically examined whether priming participants with (i.e., subtly increasing the salience of; see File S1, Note 1) the social category Black (versus White) would affect both perceptions of the relative difference in culpability between juveniles and adults and the acceptability of severe punishments for juvenile offenders who have committed serious crimes. We hypothesized that, even when they are presented with the same serious crime, people would see juvenile offenders as less different from adults and worthy of more severe punishments when exposed to an example case that included a Black American as compared with a White American. As noted, this distinction between juveniles and adults is considered foundational in the law. For example, cases that ultimately extended the protections associated with juveniles to severe crimes have hinged on this relative difference in culpability [4], [5]. At the same time, however, there are practices that may be seen as placing this distinction in jeopardy, such as assigning juveniles to adult courts for sentencing, which has been on the rise [9]. For these reasons, it is critical to understand factors that might inappropriately affect perceptions of this legal distinction, and particularly the role of race.

Contemporary social psychological research has largely focused on disparate negative outcomes occurring for the individual in the criminal justice context as a function of race: Black targets are spontaneously viewed as more criminal [10], are more likely to be assigned greater punishment [11]-[13], and even are more readily shot in “shoot-don’t shoot” computer simulations [14]. However, this research has not yet explored the effect of priming race on people’s perceptions of the foundational legal precedent establishing the difference between juveniles and adults. Given that this precedent sets boundaries on appropriate punitiveness toward juveniles, any factors that inappropriately weaken or undermine the established difference between juveniles and adults could have serious practical ramifications across the criminal justice system.

Thus, in the present study, we examined for the first time whether White Americans, a group overrepresented in jury pools [15]-[18], the legal field, and the judiciary [19] would perceive juvenile status as a mitigating factor to the same degree when primed to think of Blacks versus Whites. In other words, we asked whether race influences the extent to which juveniles are viewed as less culpable than adults and, as a result, the support for a punitive policy directed at them. Although research shows that prejudice against Black Americans is positively related to support for punitive measures [6], [20]-[23], other work also shows that the mere association of Black Americans with crime leads to greater punitiveness above and beyond the effects of explicit racial prejudice [10], [11], [14]. Thus, we predicted that priming participants with Black versus White would affect perceptions, even above and beyond the effects of differences in individuals’ level of racial prejudice.

## Methods

### Participants

A nationally-representative sample (see File S1, Note 2) of 735 White Americans participated (347 males, 388 females, *mean age*  = 50.47, *SD* = 16.51). Only those who answered the race manipulation check question correctly were included in the final, weighted sample of 658 (89.5% of the sample).

### Ethics Statement

Participants completed the study online and provided written informed consent. We attest that the data were collected in strict accordance with the ethical guidelines pertaining to the use of human subjects. The protocol was approved by the Institutional Review Board at Stanford University.

### Procedure

At the time of the study, the Supreme Court was, in fact, weighing the constitutionality of a class of penalties for juvenile defendants in non-homicide cases: life in prison without the possibility of parole. Given that the tension between protection of juvenile status and punishment fitting the crime was evident in this case, we thought it an ideal setting for investigating our hypotheses. We provided participants with factual information about this Supreme Court case. Specifically, participants read that life without parole sentences for juveniles in non-homicide cases were currently under review by the Supreme Court, and then they read some details about this Supreme Court case (e.g., that there was both support for and opposition to this sentencing option, that approximately 100 people had received life without parole sentences as juveniles for non-homicide cases). Embedded in the materials, participants read about an example recipient of this sentencing option: a 14-year-old male with 17 prior juvenile convictions on his record who brutally raped an elderly woman. This information was based on one of the two cases that the Supreme Court selected as representative for review in order to determine the constitutionality of these sentences generally [24]. We manipulated just one word across the two study conditions. In the description of the example recipient of the sentencing option, the juvenile was described as either Black or White (i.e., “a [black/white] male with 17 prior juvenile convictions…”).

#### Dependent Variables

First, we assessed participants’ general support for the sentencing option in question with the item, “To what extent do you support life sentences with no possibility of parole for juveniles when they have been convicted of serious violent crimes (in which no one was killed)?” (not at all “1” – extremely “6”). Because the legal distinction establishing that juveniles ought to be viewed as less culpable than adults is the central basis of their protected status, we then asked participants about their perceptions of how juveniles as a group should be viewed, relative to adults. Participants responded to the item, “How much do you believe that juveniles who commit crimes such as these should be considered less blameworthy than an adult who committed the same crime?” (juveniles are less blameworthy than adults “1” – juveniles and adults are equally blameworthy “6”).

#### Control Variables

Past research suggests that support for more punitive criminal policy is associated with less positive feelings toward Black Americans relative to White Americans [20] and with more conservative political ideologies [25], [26]. Although we expected these well-established factors to relate to people’s support for severe sentencing, we hypothesized that the Black race prime would influence punitiveness above and beyond the effects of these variables. Therefore, participants completed two feeling thermometer scales, rating both White and Black Americans on a scale from 0 (very cold or unfavorable feeling) to 100 (very warm or favorable feeling). We also obtained measures of political party affiliation (strong republican “1” – strong democrat “7”) and political ideology (extremely liberal “1” – extremely conservative “7,” reverse-scored) to create a political attitudes composite (α = .75). Finally, participants completed an item that probed their memory for the race of the defendant in the example case. In all analyses, we exclude those participants who did not correctly recall the race of the juvenile (see File S1, Note 3).

## Results

### Primary Hypothesis Testing

**Figure 1:**
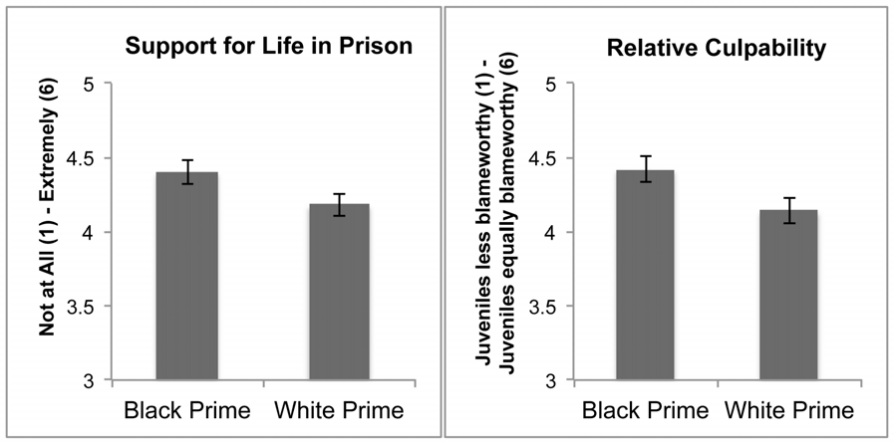
Effect of priming race on life without parole sentences and juveniles’ blameworthiness relative to adults. Participants in the Black prime condition exhibited significantly greater support for life without parole sentences and viewed juveniles’ and adults’ culpability as significantly more similar than did participants in the White prime condition. Error bars represent standard errors of the means.

Turning to our primary hypotheses, we found that participants in the Black prime condition expressed significantly more support for life without parole sentences for juveniles in non-homicide cases (*M* = 4.40, *se* = .07) than did those in the White prime condition (*M* = 4.18, *se* = .09), *t*(576.29) = 2.12, *p*<.05, Cohen’s *d* = .18 (see File S1, Note 4). Next, we examined whether associating the crime with Black Americans would also affect participants’ perceptions of an entire (legal) class of individuals: juveniles. Indeed, we found that in the Black prime condition, participants perceived juveniles as more similar to adults in blameworthiness (*M* = 4.42, *se* = .08) than they did in the White prime condition (*M* = 4.14, *se* = .09), *t*(634) = 2.33, *p* = .02, Cohen’s *d* = .19 (see Figure 1; see File S1, Note 5). Taken together, these results indicate that the association of a crime with Black (versus White) can affect both policy support and perceptions of juveniles’ culpability relative to adults.

**Figure 2:**
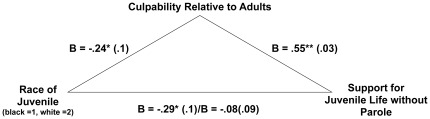
Mediational Analysis. Perceptions of the distinction between juveniles’ and adults’ relative culpability mediates the relationship between the race prime and support for juvenile life without parole sentences (in non-homicide cases). Participants in the Black (vs. White) prime condition exhibited greater (vs. lesser) support for life without parole sentences because they saw less (vs. more) of a distinction between juveniles’ and adults’ culpability.

Given these results, we then tested for mediation. First, we examined whether participants’ differential perceptions of juveniles’ culpability might mediate the effect of the race prime on support for life without parole sentences. As noted, the race prime condition predicted both support for life without parole sentences and perceptions of juveniles’ culpability relative to adults. Next, we examined whether perceptions of juveniles’ culpability relative to adults predicted support for life without parole sentences, and it did, *β* = .64, *t*(633) = 20.72, *p*<.01. Finally, we tested for mediation and found that the effect of race on support for life without parole sentencing was no longer significant, *β* = -.027, *t*(632) = .88, *p*>.3, when perceptions of blameworthiness relative to adults was included in the model, *β* = .63, *t*(632) = 20.54, *p*<.01, Sobel *z* = 2.35, *p* = .02 (see Figure 2). In other words, the degree to which participants broke down the established legal boundary between juveniles’ and adults’ culpability (applying a more adult standard of blameworthiness when the crime was associated with Black) accounted for their greater support for juvenile life without parole sentences in the Black prime condition, consistent with a mediation hypothesis.

We also explored the reverse mediation, with support for life without parole as a mediator of the link between the race prime and perceptions of juveniles’ culpability relative to adults. The effect of race on participants’ perceptions of juveniles’ culpability relative to adults was no longer significant, *β* = -.039, *t*(632) = 1.28, *p* = .2, when policy support was included in the model, *β* = .63, *t*(632) = 20.54, *p*<.01, Sobel *z* = 2.15, *se* = .038, *p* = .03. Although this was also significant, we believe the former mediational model is more plausible both because the former model is more consistent with previous research [11] and because the effect of condition was reduced to a greater degree. In the former model (with perceptions of relative culpability as the mediator), the effect of condition on policy support was reduced to β = -.027. In the latter model (with policy support as the mediator), the effect of condition on perceptions of relative culpability was only reduced to β = -.039. Nevertheless, in future studies, researchers should continue to investigate this process to determine the conditions under which judgments of culpability drive the effect of race on punitiveness.

### Control Variables

Although past work has shown some differences in punitiveness by gender [13], we found no main effects of participant gender on our target dependent variables. We should note, however, that the results are unchanged even controlling for gender. Participants’ political attitudes did not differ by condition (Black prime condition mean = 3.76, *SD* = 1.58, White prime condition mean = 3.81, *SD* = 1.81, p>.5), but were significantly correlated with the key dependent variables (support *r* = -0.27, *p*<.01; culpability *r* = -0.26, *p*<.01). We next examined participants’ ratings of how warm or cold they felt toward Black and White Americans. Paired samples t-tests revealed that participants in both conditions rated themselves as warmer toward White than Black Americans: in the Black prime condition, *M_White* = 72.81, *se* = .93, vs. *M_Black* = 62.53, *se* = 1.05; *t*(289) = 11.43, p<.01, Cohen’s *d* = 1.3; in the White prime condition, *M*_*White* = 72.8, *se* = .95 vs. *M*_*Black* = 61.21, *se* = 1.03; *t*(342) = 11.96, *p*<.01, Cohen’s *d* = 1.3. The degree of this bias did not differ by condition (*p*>.2). Moreover, replicating past research [20], we also found that warmth toward Black Americans correlated in the predicted direction with both dependent variables: less positive feelings toward Black Americans were associated with greater support for life without parole (*r* = -0.17, *p*<.01), and were associated with perceptions of juveniles’ culpability as closer to that of adults (*r* = -0.14, *p*<.01). Warmth toward White Americans did not correlate with either of the dependent variables (*p*s>.4). When controlling for both political attitudes and warmth toward Black Americans, the effect of the manipulation on support for severe sentencing and perceptions of juveniles’ blameworthiness relative to adults remained significant, as did the mediational analysis. Thus, the effect of a subtle race prime on support for punitive policy and perceptions of juveniles’ culpability functioned above and beyond the effects of political ideology and warmth toward Black Americans. We also explored whether either political attitudes or warmth toward Black Americans interacted with condition to affect the dependent variables, but neither did. These results indicate that the effect of priming race on support for severe sentencing and perceptions of juveniles’ blameworthiness relative to adults was the same for more liberal vs. more conservative participants and even for participants who exhibited more vs. less warmth toward Black Americans.

## Discussion

A one-word priming manipulation affected people’s support for the most severe punishment available for juveniles and their perceptions of the distinction between juveniles and adults. Moreover, the degree to which the legal difference between juveniles’ and adults’ culpability was undermined may have accounted for how much more support for life in prison without the possibility of parole people exhibited in the Black prime (vs. White prime) condition, although given the reverse mediation this result bears additional investigation. These results extend past social psychological studies showing that race affects individual target’s outcomes. Here, we illustrate that the application of important legal policies are subject to such inappropriate influence as well. As such, the present research provides the first direct empirical evidence that a racial priming manipulation can affect the degree to which juveniles (in general) are afforded the established protections associated with their age status in the context of a severe crime.

Although the effect sizes were modest [27], the manipulation was particularly subtle – a single word prime embedded in a passage of text – and the outcome particularly consequential – whether juveniles would be eligible for life in prison or not. If the salience of Black Americans were multiplied, as is likely the case in crime-relevant contexts [10], then might the consequences of this salience likewise be magnified? Indeed, the present research raises the possibility that being primed over and over through exposure to Black individuals or racially coded language could produce changes in judges’ and juries’ perceptions of culpability and their ensuing punitive judgments. This would have troubling implications for juvenile justice more broadly because it suggests that juvenile status may be more fragile than previously considered – vulnerable to being undermined in any extreme case and subject to differences based upon the racial associations salient in the moment.

As noted, juvenile justice policies appear to have been largely protected from the general increasing punitiveness associated with adult criminal justice in the U.S., and these protections have been extended to include severe offenses [4], [5]. In contrast, the current results indicate that juvenile justice contexts may not be protected from the inappropriate influence of race, a factor that has been well-established as an obstacle to equal application of the law in adult court contexts [3], [7], [10]. These results also emphasize that the influence of race in the justice system can extend beyond unfair outcomes for individual targets to encompass the policies that are instituted and ultimately applied to all. If racial associations are made salient in the contexts where people exhibit public support for crime policies [22] and if these associations affect beliefs about the nature of juveniles, as suggested here, the juvenile justice context may come to achieve the same increasing punitiveness that has been evident in the adult justice context. Thus, the current research identifies ways in which the legal protections associated with juvenile status may be more fragile than previously considered.

The Supreme Court determination that juveniles ought to be considered distinct from adults was informed by evidence and expert opinion from the fields of psychology and neuroscience that highlighted differences in the cognitive ability, neurological development, and reasoning skills of juveniles vs. adults [4], [5]. As important as this research was for determining policy that established the juvenile-adult distinction, none of the findings addressed people’s subjective perceptions of the difference between juveniles and adults or how these perceptions might be shaped by race. Thus, the present study augments this previous literature by examining people’s views of the distinction between juveniles and adults and by showing that this distinction is undermined in the context of even a single Black (vs. White) example case. The results also extend the established literature in social psychology examining the cognitive association between the social category “Black” and criminality [10], and raise the possibility that this race-crime association may be at odds with lay people’s typical notions about the innocence of juveniles. As a consequence, when Black Americans are salient, differences in people’s perceptions of the juvenile-adult distinction could have meaningful effects in the criminal justice system, potentially tipping the scales when the severity of an offense is at odds with the protections associated with juvenile status. Future research should continue to examine the role of race in judgments that determine whether juveniles as a group are given the full protection of the law.

## Supporting Information

File S1 Supporting information file with footnotes.
(DOC)
