# Supplementary material for: Race and the Fragility of the Legal Distinction between Juveniles and Adults
Source: PLoS One. 2012 May 23;7(5):e36680. doi: 10.1371/journal.pone.0036680 (PMC3359323; doi:10.1371/journal.pone.0036680)
Supplement: File S1 — Supporting information file with footnotes. (DOC) [file pone.0036680.s001.doc]

**Supplementary File 1**

Note 1: Priming is a standard procedure in psychology used to increase the accessibility of a concept. Research has shown that subtly increasing the salience of a social category can lead to later perceptual and behavioral changes in a perceiver [28].

Note 2: Data collected by Time-sharing Experiments for the Social Sciences, NSF Grant 0818839, Jeremy Freese and Penny Visser, Principal Investigators.

Note 3: This removes 13.33% of the sample. Comparing the accuracy across conditions, we found that 13.95% of participants in the White prime condition failed to answer the race manipulation check question correctly, versus only 4.93% in the Black prime condition, χ2(1, 702)= 16.95, p < .01. This result suggests that it might have been easier for participants in the Black prime condition to encode the race of the defendant, perhaps providing evidence of a broader cultural association between Black Americans and crime [10] in the juvenile context.

Note 4: The assumption of homogeneity of variances was violated in this analysis, Levene’s Test for Equality of Variance *F* = 4.42, *p* < .05. Therefore, the degrees of freedom and *t-*test statistics were computed not assuming homogeneity of variance.

Note 5: When we compare the overall sample mean for blameworthiness relative to adults to the endpoint of the scale labeled “juveniles and adults are equally blameworthy,” the sample mean is significantly less than this endpoint, one sample t-test, t(635) = -28.51, p < .01, Cohen’s d = 2.26. This indicates that, across the two conditions, juveniles are still viewed as significantly less culpable than are adults. However, as the results illustrate, the degree to which juveniles are viewed as less culpable is significantly predicted by the race prime condition.

References

28. Bargh J A, Chen M, Burrows L (1996) Automaticity of social behavior: Direct effects of

construct and stereotype activation on action. *Journal of Personality and Social Psychology, 71*: 230-244.
